# Breast cancer stem cells: tools and models to rely on

**DOI:** 10.1186/1471-2407-9-202

**Published:** 2009-06-25

**Authors:** Emmanuelle Charafe-Jauffret, Christophe Ginestier, Daniel Birnbaum

**Affiliations:** 1 Centre de Recherche en Cancérologie de Marseille, Laboratoire d'Oncologie Moléculaire, UMR891 Inserm/Institut Paoli-Calmettes, Marseille, France

## Abstract

There is increasing evidence for the "cancer stem cell (CSC) hypothesis", which holds that cancers are driven by a cellular component that has stem cell properties, including self-renewal, tumorigenicity and multi-lineage differentiation capacity. Researchers and oncologists see in this model an explanation as to why cancer may be so difficult to cure, as well as a promising ground for novel therapeutic strategies. Given the specific stem cell features of self-renewal and differentiation, which drive tumorigenesis and contribute to cellular heterogeneity, each marker and assay designed to isolate and characterize CSCs has to be functionally validated. In this review, we survey tools and markers available or promising to identify breast CSCs. We review the main models used to study breast CSCs and how they challenge the CSC hypothesis.

## Background

Understanding the role of CSCs during carcinogenesis, from tumor initiation to metastasis formation, has become a major focus in stem cell biology and in cancer research. Considerable efforts are directed towards developing clinical applications of the CSC concept. However, it is crucial to functionally validate each marker and model utilized to isolate and characterized CSC. In this review, we give an overview of the tools available to study breast CSCs and describe their implications to improve breast cancer treatment. The cancer stem cell (CSC) model holds that tumors are organized in a cellular hierarchy in which CSCs are the only cells with unlimited proliferation potential and with the capability of driving tumor growth and progression. According to this model, cancers originate from the malignant transformation of an adult stem cell or progenitor through the disregulation of the normally tightly regulated self-renewal program. This leads to clonal expansion of stem/progenitor cells that undergo further genetic or epigenetic alterations to become fully transformed. As a consequence of this, tumors contain a cellular component of CSCs which retain the key stem cell properties that initiate and drive carcinogenesis.

A major goal of both researchers and oncologists is to understand how many and which tumor cells must be eliminated for a given treatment to succeed. Eliminating cancer cells that have limited proliferation potential, while sparing cancer stem cells that have unlimited proliferation potential will eventually result in relapse or recurrence. This hypothesis has been recently validated by different studies that described CSCs from various tissues as a cell population resistant to current anticancer therapies, antimitotic agents and radiation [1-6].

Some years ago, a subset of cells from a neuroblastoma cell line identified upon their capacity to exclude vital dye as a Side Population (SP cells) showed the ability to resist chemotherapy. Although SP and non-SP cells were able to proliferate in the absence of antimitotic agents, SP cells could proliferate as colonies in the presence of mitoxantrone, while non-SP cells could not. These data suggested that a subpopulation of neuroblastoma cells shared some properties with stem cells and were selected *in vitro *by chemotherapy [7].

In breast tumors, the use of neoadjuvant regimens showed that conventional chemotherapy could lead to enrichment in CSCs in treated patients as well as in xenografted mice [1,3]. Furthermore, primary mammospheres from chemotherapy-treated patients showed similar mammosphere-initiating capacity after eight to ten generations, whereas cells from untreated patients vanished within two to three generations, suggesting again an increase in cells with self-renewal potential after chemotherapy [3]. The radiation effect on CSCs was studied *in vitro*, using staining of phosphorylated histone H2AX and the measurement of reactive oxygen species as functional tests for radiation resistance [2]. In MCF7, CSC/progenitors isolated as mammospheres were more resistant to radiotherapy than cells in monolayer culture, and fractionated radiotherapy increased the proportion of breast CSCs with the CD44^+^/CD24^-/low ^phenotype [2].

These data reinforce the belief that CSCs resist many current therapies and that they are the actual targets to eliminate if treatment is to be curative. Interestingly, treatment of ERBB2-positive tumors with the EGFR/ERBB2 inhibitor lapatinib led to a small decrease in the percentage of breast CSCs [1]. Thus, appropriate targeted therapies can eliminate CSCs.

The success of our efforts in translating cancer stem cell research into clinical practice depends on how thorough and rigorous we are at characterizing these cells. It also relies on how practical and reliable are the markers and assays designed to study CSCs.

## Techniques for the characterization of cancer stem cells

### Side population technique

The SP technique has been used for many years to isolate both normal and tumor stem cells from different organs and species [7-10]. It is based on the abilities of stem cells to exclude vital dyes. Normal and cancer stem cells express transmembrane transporters, such as the ATP-binding cassette protein, ABC transporter ABCG2/BCRP1 (breast cancer resistance protein 1). These molecules exclude dyes such as Hoechst 33342 or Rhodamin 123 from the cells, a property not found in differentiated cells that remain positive for the dye.

SP cells isolated from reduction mammoplasty of normal breast in volunteer healthy women, were found to express more BCRP1 than non-SP cells. Moreover, a specific BCRP1 inhibitor (Ko143) reduced SP formation, suggesting that BCRP1 confers the SP phenotype in mammary epithelial cells. Interestingly, SP cells did not express either luminal or myoepithelial markers (EMA and CALLA/CD10) or estrogen receptor (ER), whereas non-SP cells did [11]. A relatively undifferentiated phenotype without any luminal marker expression was also displayed by cells from mammospheres, obtained after non-adherent serum-free culture of normal mammary gland, and proven to be enriched in stem/progenitors cells [12]. The SP fraction from uncultured mammary cells represented ~1% of cells. In contrast, in mammospheres, the SP fraction represented 27% of the cells and could generate bi-lineage colonies when cultured under differentiating conditions, suggesting that the SP fraction contained the bipotent progenitors and was capable of mammosphere formation [12].

In the mouse, SP cells have been shown to regenerate the gland upon transplantation [13] and to express genes encoding putative stem cell markers such as α6-integrin and telomerase [14]. A SP isolated from the breast cancer cell line MCF7 was found to represent 2% of the total cell line. It contained the tumorigenic fraction from MCF7, as demonstrated by transplantation experiments in NOD/SCID mice xenografts. This fraction was also able to reconstitute the initial heterogeneity of the cell line [15,16].

The SP technique for CSCs has also been successfully used in other species and tissues [7-10,17]. However, functional studies using Hoechst staining are limited by the toxicity of this agent. Consequently, if Hoechst-positive cells do not grow *in vivo *or *in vitro*, the reason could be a direct toxic effect of the dye, shedding doubts on the reliability of the experiments. Furthermore, evidence from mouse models indicates that the mammary repopulating units with functional stem cell activity are not contained within the SP [9,18]. This is mainly why the SP technique is no longer the preferred approach for stem cell studies.

### Expression of cell surface markers

Expression of cell surface markers has been widely used to isolate stem cells, but the choice of marker can greatly vary depending on tissues or species. The following markers have been used in the study of breast stem cells:

#### -CD44+/CD24-^//low ^lin^-^

The pioneering study by Clarke and colleagues used breast cancer xenografts to isolate a population of cells able to initiate tumors in NOD/SCID mice [19]. This population was defined by the combined expression of cell surface markers CD44^+^/CD24^-/low^/lin^-^. As few as 200 of these cells generated tumors in NOD/SCID mice whereas 20,000 cells that did not display this phenotype failed to do so. The NOD/SCID tumors recapitulated the entire heterogeneity of the initial tumor. Furthermore, the CD44^+^/CD24^-/low^/lin^- ^cell population was able to reinitiate tumors in NOD/SCID mice, and retained this ability after serial passages. Thus, these cells, which were able to self-renew, to differentiate, and displayed tumorigenic capacity, had CSC features.

The CD44^high^/CD24^low ^phenotype has been used to isolate stem cells from the human normal mammary epithelium. This phenotype seems to be conserved during the carcinogenesis process and thus is an important tool to study breast cancer progression, however, it is limited by the great cellular heterogeneity of the CD44^+^/CD24^-/low^/lin^-^population, which probably does not contain solely *bona fide *CSCs. The use of the CD44^+^/CD24^-/low^/lin^- ^phenotype and another marker, the ALDEFLUOR assay, which measures the Aldehyde dehydrogenase enzymatic activity, demonstrated that cells able to initiate tumor in mice were within the ALDEFLUOR-positive cells, the cells displaying both phenotypes being the most tumorigenic, and that none of the CD44^+^/CD24^-/low^/lin^- ^cells without ALDEFLUOR activity could grow in mice [20]. These results indicate that the CD44^+^/CD24^-/low^/lin^- ^population contains some but not all the CSCs in breast tumors. Moreover, while CD44 appears to be a common stem cell marker [21-23], as well as a promising therapeutic target [24,25], the CD44^+^/CD24^-/low^/lin^- ^phenotype is probably tissue-restricted. Pancreatic cancer cells with stem cell properties of self-renewal, ability to produce differentiated progeny, and increased expression of the developmental signaling molecule sonic hedgehog, display a CD44^+^/CD24^+^/ESA^+ ^phenotype [26,27].

#### - CD49f/ITGA6/α6-integrin

A combination of cell surface markers was used to purify a rare subset of adult mouse mammary stem cells that were able individually to regenerate an entire mammary gland within six weeks *in vivo*. These cells, sorted as CD45^-^/Ter119^-^/CD31^-^/Sca-1^low^/CD24^med^/CD49f^high^, were designated as mammary repopulating units (MRUs) and expressed markers associated with basal cells (smooth muscle actin and keratin 14). They were different from progenitor cells that represented the mammary colony forming cells and expressed keratin 8, 18 and 19 transcripts and keratin 6 protein [28].

Interestingly, a recent study described a subpopulation that overexpressed the α6-integrin in the human breast luminal cell line MCF7. This population presented cells capable of growth in anchorage-independent conditions as spherical organoids. These cells displayed resistance to pro-apoptotic agents and greater tumorigenicity than the whole cell line, with as few as 1,000 cells able to form tumors in immunodeficient mice [29]. Moreover, knockdown of α6-integrin/ITGA6 caused mammosphere-derived cells to lose their ability to grow as mammospheres and abrogated their tumorigenicity in mice, proving that ITGA6 is required for the growth and survival of this highly tumorigenic subpopulation of cancer cells, and suggesting that this adhesion molecule is a potential therapeutic target.

#### - CD133/PROM1/prominin

The difference in cellular hierarchy between estrogen-receptor (ER)-negative and ER-positive tumors has only been addressed recently using cleared fat pad transplantation assay in mouse and flow cytometry analysis [30]. The mouse mammary epithelium contained three distinct cell populations: basal/myoepithelial cells, defined by the CD24^low ^phenotype, and two distinct CD24^high ^luminal epithelial populations with different prominin-1 expression. The CD24^high^/prominin-1^+ ^cells belonged to the luminal compartment. The majority of stem/progenitor cell activity in the adult virgin mouse mammary epithelium was found to be located in the basal compartment, confirming and extending previous observations [30]. In contrast, the ER-positive luminal compartment contained little *in vivo *stem/progenitor cell activity, indicating that the hormone-sensing and *in vivo *stem/progenitor activities of the mammary epithelium are properties of distinct, separate cell populations.

Such a clearcut distinction has not yet been firmly established in the human mammary gland. However, a model proposes that the steroid receptor-positive population is a slowly dividing stem cell population whereas the adjacent proliferative cells represent transient amplifying populations [31]. Disregulation of normal self-renewal results in increased symmetrical cell divisions of stem cells, and could explain the low proliferation rate of steroid receptor-positive cells in both pre-cancerous and cancerous breast lesions.

CD133 might be a good candidate to explore carcinogenesis and estrogen-dependent tumor progression. In cell lines from mammary tumors *Brca1 *knockout mice, the expression of CD133 cells is associated with stem cell properties as well as CD44^+^/CD24^-/low ^phenotype [32]. However, the use of prominin has never been as successful in isolating CSCs from human breast cancer as it has been in other organs such as brain tumors or colon carcinomas [33-35]. In any case, testing the efficiency of CD133 expression to isolate CSCs from luminal ER-positive breast tumors remains an interesting point to explore.

#### - CD29/β1-integrin and CD61/β3-integrin

CD29 and CD61 are two cell surface proteins that may play a role in luminal cell fate determination. CD29 distinguishes two distinct mammary epithelial populations among immature CD24^+ ^cells, one enriched for luminal (CD29^low^/CD24^+^) and one for mammary stem cells (CD29^high^/CD24^+^)[36]. The luminal CD29^low^/CD24^+ ^subpopulation can be further subdivided using CD61 into CD61^+ ^luminal progenitors and CD61^- ^mature luminal cells [37]. Moreover, in the mouse model of luminal MMTV-WNT1 tumors, flow cytometry sorting upon CD61, identified a CSC population highly enriched for tumorigenic capability compared to CD61^- ^cells [38]. CD29 and CD61 could therefore be promising targets for human clinical applications based on data obtained from normal and tumoral mouse mammary gland studies.

Flow cytometry methods using cell surface markers have been successfully applied to mice and human samples to isolate stem cell populations. Markers available for cell sorting of stem cell populations of the mouse mammary gland are numerous, the functional assays well validated and the cellular hierarchy partly established. In contrast, in the human mammary gland, the markers are scarce, the assays difficult to standardize, and the actual hierarchy remains to be defined. Furthermore and curiously, the markers used in mice to sort specific stem cell populations are rarely valid in human. This observation emphasizes the need for other, more "universal" assays.

### ALDEFLUOR assay

The ALDEFLUOR assay may fit the universality required for a stem cell marker to be reliable across species and tissues. It is based on the enzymatic activity of aldehyde dehydrogenase 1 (ALDH1), a detoxifying enzyme responsible for the oxidation of retinol to retinoic acid. ALDH1 may have a role in early differentiation of stem cells [6,39]. High ALDH1 activity is associated with several types of murine and human stem hematopoietic and neural stem and progenitor cells [40-43]. As few as 10 ALDEFLUOR-positive cells isolated from the rat hematopoietic system are capable of long term repopulation of bone marrow upon transplantation in sub-lethally irradiated animals [40]. ALDH1 activity also identified CSCs in multiple myeloma and leukemia patients with high capability of engraftment into NOD/SCID mice [43,44]. A recent study showed that ALDEFLUOR-positive cells isolated from the mouse brain were capable of self-renewal and able to generate neurospheres and neuroepithelial stem-like cells. These cells were capable of differentiation into multiple cell lineages *in vitro*, generating neurons and glia in culture. Furthermore, ALDEFLUOR-positive cells had a higher capacity to engraft *in vivo*, upon transplantation in brain, compared to ALDEFLUOR-negative cells [45].

This method has been recently used with success to isolate stem and progenitors cells from mammary tissues. ALDEFLUOR-positive cells isolated from both normal and tumoral human breast had phenotypic and functional characteristics of mammary stem cells. Furthermore, the ALDEFLUOR-positive population isolated from human breast tumors contained the CSC population as demonstrated by the ability of these cells but not of ALDEFLUOR-negative cells to generate tumors in NOD/SCID mice. Serial passages of ALDEFLUOR-positive cells generated tumors recapitulating the phenotypical diversity of the initial tumor [20].

However, the ALDEFLUOR assay does have some limitations for the isolation of the most tumorigenic population, notably in tumors of different origin. For example, both ALDEFLUOR (bright) and ALDEFLUOR (low) from the lung carcinoma cell line H 522 were able to initiate tumors after inoculation into NOD/SCID mice. Moreover, tumors generated from ALDEFLUOR (low) cells grew faster and bigger than the tumors from ALDEFLUOR (bright) and this remained true among passages. These results suggest that the ALDEFLUOR-positive population in lung carcinoma is not stem cell-enriched compared to the ALDEFLUOR-negative population [46]. Furthermore, the stem cell population identified using the ALDEFLUOR assay is probably heterogeneous, and needs to be dissected using additional markers. In breast cancer cell lines, the ALDEFLUOR-population has been divided by the use of CD44^+^, CD24^- ^and CD133. ALDEFLUOR-positive CD44^+^/CD24^- ^and ALDEFLUOR-positive CD44^+^/CD133^+ ^populations displayed the greatest tumorigenic and metastatic potential. This is the first time that CSCs obtained with a given marker are further divided using additional markers into distinct metastatic or not metastatic subpopulations [47]. In human hematopoietic stem cells, the ALDEFLUOR^high ^lin^- ^population has also been separated in CD133-positive and negative subsets, with the former showing enhanced repopulating capacity in recipients of serial, secondary transplants [42].

These data open new fields for functional stem cell studies with therapeutic applications using marker combinations.

### *In situ *detection

*In situ *detection of stem cells has the potential to transfer stem cell quantification to routine clinical practice for patient treatment and prognosis evaluation. It also allows the determination of the CSCs' location within the tumor-either primary or metastatic sites-, and the detection of stem cells in pre-invasive stages as well as their modifications during pre-malignant to malignant progression.

Aldehyde dehydrogenase activity has been mostly attributed to the function of aldehyde dehydrogenase1A1, one of the main ALDH cytoplasmic isoform. This enzyme is highly expressed in stem cells and is thought to regulate stem cell function [6]. *In situ *immunostaining of ALDH1A1 has been measured in formalin-fixed, paraffin-embedded breast tumors and it identified both normal and malignant human mammary stem cells; 30% of the breast tumors analyzed presented a relatively small ALDH1-positive cell population. The analysis of ALDH1 expression in human breast carcinomas showed that the expression of this stem/progenitor cell marker is a powerful predictor of poor clinical outcome [20].

These data suggest that *in situ *detection of CSCs using ALDH1 immunohistochemistry is a valid, simple method and a powerful prognosis marker. However, the use of ALDH1A1 to detect stem cells is not free of controversy. In transgenic mice, ALDH1A1 deficiency did not affect hematopoiesis and hematopoietic stem cell (HSC) function, or ALDEFLUOR staining [48]; it is possible that other isoforms of ALDH, notably cytoplasmic isoforms such as ALDH1A3 were responsible both for the maintenance of HSC function and the remaining ALDEFLUOR staining.

Another method used to detect stem cells *in situ *is double immunostaining using CD44 and CD24 antibodies and subsequent quantification of the CD44^+^/CD24^low/- ^phenotype. The prevalence of CD44^+^/CD24^low/- ^cells in paraffin-embedded tumors was lower than 10% in the majority of cases, and this phenotype was neither associated with clinico-pathologic characteristics nor with clinical outcome [49]. Strikingly, the presence of the CD44^+^/CD24^low/- ^tumor cells was inversely associated with lymph node metastasis (P = 0.019) and tended to be inversely associated with the stage of the disease (P = 0.068) [50]. Another study on paraffin-embedded breast tumors revealed that the CD44^+^/CD24^low/- ^phenotype is most common in the basal subtype and particularly common in *BRCA1 *hereditary tumors, of which 94% contained CD44^+^/CD24^low/- ^cells. The CD44^+^/CD24^low/- ^phenotype was surprisingly scarce in ERBB2-positive tumors, which had a predominantly CD24+ status [51]. In bone marrow specimens of 50 patients with early breast tumors, CD44^+^/CD24^low/- ^phenotype in cytokeratin-positive cells was detected in 72% of all disseminated tumor cells (DTC) compared with less than 10% in primary tumors [52]. In all these studies, if the CD44^+^/CD24^low/-^phenotype is associated with the basal subtype, or appears in DTC as a first step toward metastasis, there was no correlation with clinical outcome, whether disease-free, relapse-free, metastasis-free or overall survival.

Even if the CD44^+^/CD24^low/- ^phenotype is a valuable marker for the isolation of breast CSCs it cannot be used in clinical settings. As pointed out by Gabriela Dontu in a recent commentary, the use of these markers raises several important questions [53]. Is the CD44^+^/CD24^low/- ^phenotype associated with CSCs only in certain breast cancers, predominantly basal-like or BRCA1? Are cancers that do not contain cells with this phenotype driven by a different CSC? If this is the case, do the CSCs not bearing the CD44^+^/CD24^low/- ^phenotype have a different origin? Is it possible that the CD44^+^/CD24^low/- ^phenotype is a dynamic one, i.e. can it be lost and *de novo *acquired during tumor progression, as a result of genetic instability and epigenetic changes? Thus, a marker valuable to isolate CSCs may not be applicable in the clinic, demonstrating all the pitfalls that come with the validation process.

**Figure 1:**
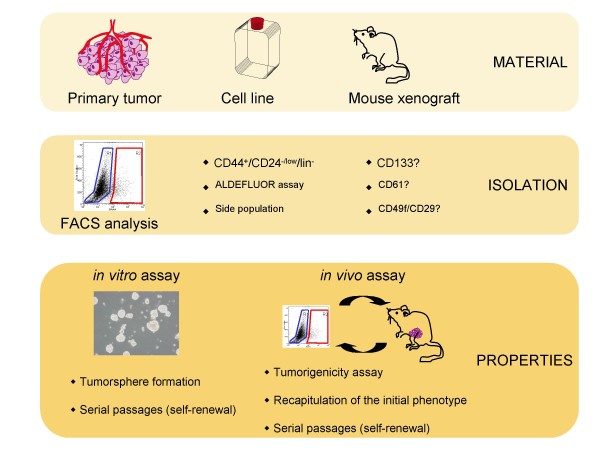
**Markers and model for breast cancer stem cell studies**. The main assays, markers and models used to study breast cancer stem cells are schematically represented. Models and assays rely on the main stem cells properties that are self-renewal ability and differentiation potential. The various markers illustrate the great phenotypic diversity of the cancer stem cell population.

In order to progress in this field, we will need to further characterize the breast CSC population using validated methods. Among the different methods utilized to isolate breast CSCs some are promising but require validation with a combination of two or more methods to improve our knowledge on the cells that drive tumor growth and evolution (Figure 1). To better understand how these cells sustain the growth and expansion of the tumor, and because the stem cell component within primary tumors can be scarce, it is necessary to develop convenient and valid models/systems for the study of stem cells, with the final aim of developing therapeutic strategies.

### Anchorage-independent cell culture

Cell culture in non-adherent conditions was initially adapted to normal breast obtained from reduction mammoplasty. Human mammary stem and progenitor cells were able to survive in suspension and produce spherical colonies (mammospheres) composed of both stem and progenitor cells. These non-adherent mammospheres were enriched in early progenitor/stem cells and able to differentiate along the three mammary epithelial lineages and to clonally generate complex functional structures in reconstituted 3D culture systems as well as reconstitute human normal mammary gland in mice [12]. This system is now widely used to study underlying mechanisms of growth under anchorage-independent conditions, and by extension, to discover pathways implicated in stem/progenitor cells survival.

Hedgehog pathway, BMI1/polycomb and NOTCH signaling have been shown to play a critical role in normal human mammary development by acting on both stem cells and progenitor cells, affecting self-renewal and lineage-specific differentiation [54,55]. Furthermore, aberrant activation of NOTCH signaling is involved in early breast tumorigenesis. Increased NOTCH intracellular domain staining was found associated with tumor recurrence at five years (P = 0.012) [56]. Confirming aberrant NOTCH signaling in early stages of the disease, the formation of mammospheres from primary pre-invasive ductal carcinoma *in situ *(DCIS) was reduced when NOTCH signaling was inhibited using either DAPT, a γ-secretase inhibitor, or a NOTCH4-neutralizing antibody [56].

The mammosphere assay, based on the unique property of stem/progenitor cells to survive and grow in serum-free suspension, was also successfully used to establish long-term cultures enriched in stem/progenitor cells from invasive tumor samples. The mammospheres formed in these conditions were called tumorspheres. They showed an increase in SP fraction and in CD44^+^/CD24^-//low ^cells, overexpressed neoangiogenic and cytoprotective factors, expressed the putative stem cell marker OCT4, and displayed high tumorigenic potential in NOD/SCID mice [57].

Thus, the development of *in vitro *suspension culture systems not only provides an important new tool for the study of mammary cell biology, but also has important implications for understanding key molecular pathways in both normal and neoplastic stem cells.

## Resources for the characterization of cancer stem cells

### Breast cell lines

Cell lines have been widely used for decades in the study of cancer. Since most of the recurrent alterations present in a primary tumor are retained in the corresponding cell line, this system has provided invaluable insights into the role played by deregulated genomic and transcriptional pathways in mammary oncogenesis [58].

Whether cell lines retain the hierarchical organization of the primary tumors they derived from is an important question that has been recently challenged in relation to cell lines from various origins such as brain glioma, breast cancer, ovarian cancer, lung cancer, prostate cancer, head and neck carcinoma, neuroblastoma, and Ewing sarcoma [7,15,59]. CSCs have also been isolated from different mouse and human breast cancer cell lines. Recently, a study reported the isolation of a putative CSC population in cell lines derived from Brca1Δ^exon11^/P53^+/-^mouse mammary tumors. This population expressed stem cell markers (CD44^+^/CD24^-/low ^or CD133^+^) and had the ability to grow as spheres, to repopulate *in vitro *the parental cell fractions after several passages, to resist chemotherapeutic agents, or to be more tumorigenic than the parental line [32].

A subpopulation of MCF7 was able to grow as spherical organoids in anchorage-independent conditions, displayed resistance to pro-apoptotic agents and greater tumorigenicity than its parental line in immunodeficient mice [29]. Several other studies have used putative CSC markers such as CD44^+^/CD24^-/low ^to identify similar populations within breast cancer cell lines, but given that CD44 is a basal marker, this phenotype did not isolate the tumorigenic population [60]. While these studies represent a major advance in validating the use of cell lines for stem cell biology, they lack important key features such as *in vivo *validation of self-renewal ability and differentiation, or are restricted to one or few cell lines.

In a recent study, we used the ALDEFLUOR assay to isolate and characterize CSCs from 33 breast cell lines, derived from normal and malignant mammary tissue [61]. 23 of the cell lines contained an ALDEFLUOR-positive population that displayed stem cell properties *in vitro *and in NOD/SCID xenografts. Gene expression profiling identified a 413-gene "cancer stem cell" profile that included genes known to play a role in stem cell function as well as genes such as CXCR1/IL8RA not previously reported to play such a role. Recombinant IL8 increased mammosphere formation and the ALDEFLUOR-positive population in breast cancer cell lines. We further showed that ALDEFLUOR-positive cells are responsible for mediating metastasis. These studies confirm the hierarchical organization of immortalized cell lines.

The use of cell lines can facilitate the characterization of regulatory pathways of cancer stem cells and identify potential stem cell markers and therapeutic targets.

### Human xenograft models

Many different pathways are involved in the determination of stem cell fate. These may be deregulated in cancer and hence represent potential pathways to target with new therapeutic strategies. Molecules that target these pathways can be first tested *in vitro *but they need to be subsequently validated *in vivo *in immunodeficient animals. Despite the caveats represented by a change in the functional properties of CSCs in the animal host and the changes in the niche (tumoral stroma, hormonal influence), the xenograft model of patient samples appears to be the closest experimental system to tumors in human patients.

Unlike cell line-derived xenografts, tumor xenografts maintain the cell differentiation and morphology, the architecture, and molecular signatures of the original tumors [62]. Vasculature, stroma, central necrosis, and peripheral growth occur in tumor-bearing mice in a way that is similar to that of the patient's tumor. Furthermore, tumor xenografts are the most relevant way to test CSC properties such as the ability to form tumors, self-renewal potential and capacity to differentiate. Among the large variety of tumors transplantable into immunodeficient mice, breast cancers are among the most difficult to establish [63].

When introducing tissue fragments or dissociated human mammary epithelial cells (hMECs) into the cleared mouse mammary fat pads of immunodeficient mice to recreate a functional gland, the microenvironment plays an eminent role [64]. Early attempts were unsuccessful presumably because of the inadequate stromal environment of the mouse mammary fat pad. Success came when normal fibroblasts were introduced in the fat pad of NOD/SCID mice prior to injection ("humanization"). Human primary mammary cells were able to survive and colonize the humanized mammary gland [65]. Orthotopic injection of epithelial normal or tumoral cells in cleared and "humanized" fat pad is closer to the human tumor and more suitable for stem cell studies compared with subcutaneous injection [20].

However, concerns about the adequacy of immunodeficient mice model for stem cell studies remain. A report has recently challenged the use of immunodeficient mice to transplant human cancer cells [66]. In this study, as few as 10 leukemic cells from mice genetically engineered to develop leukemia were injected into genetically compatible healthy animals. All recipient mice developed leukemia, raising concerns that the experiments do not accurately reflect cancer initiation and progression in human patients. Results suggested that the mouse does not provide the right environment for the growth of human cancer cells, and questioned the CSC hypothesis. More recently, it was shown that the frequency of tumor-initiating cells detected in human melanoma was highly dependent on the mouse model used for their assessment [67]. This frequency was as high as one in four cells when assayed in highly immunodeficient NOD/SCID Il2rg^-/- ^mice but several orders of magnitude lower in NOD/SCID mice (that have NK cells). The too high frequency of tumor-initiating cells questioned the CSC model.

Another interpretation of data suggests that the amount of tumor-initiating cells is not automatically small but can vary among tumors. The CSC hypothesis does not rely on the size of the cancer-initiating population but on the presence of a hierarchy within the tumors. Moreover, the use of re-transplantation of marked cell populations allows for the distinction of self-renewal from proliferation, a property of many cell types. In contrast, because Quintana *et al. *did not utilize defined cell populations [67], the demonstration of self-renewal would require tumor generation in serial passages of single cells. The increase in the number of tumor-initiating cells in a more permissive environment may be an argument in favor of environment-dependent properties for these cells. Nevertheless, these results highlight the major role of the stroma/microenvironment in CSC studies, and argue in favor of the choice of a xenograft model as close as possible to the native human environment.

## Conclusion

The application of the cancer stem cell model to the clinic implies that the elimination of CSCs is mandatory to cure cancer. Because normal and tumor stem cells often share common pathways, the use of drugs that target specific stem cell pathways is both a powerful strategy and a great challenge. In this context, it appears more significant than ever to use reliable tools and systems for stem cell studies. Different markers have been described to isolate and target stem cells, but a striking feature is that there is relatively little overlap between the different CSC markers reported in different tumor types or species (human, mice).

It is essential to define if and how solid tumors are formed from a single CSC and to determine whether CSCs are heterogeneous and exhibit different degrees of "stemness". Analysis of patients at different stages of disease, and specially a follow-up of CSCs during and after treatment or at the time of relapse will help answer the question of clonal evolution and is therefore a major goal in order to adapt treatment accordingly.

While it is now well established that the CSC component is the target to aim for treatment, it remains to be determined whether it is a unique target, and what is the best way to counteract its potential adaptation to the host environment: progression, expansion and response to treatment.

## Abbreviations

CSC: Cancer stem cell; DTC: Disseminated tumor cell; DCIS: Ductal carcinoma in situ; HSC: Hematopoietic stem cell; Human mammary epithelial cell; NK: Natural killer; NOD/SCID: Non-obese disease/severe combined immunodeficiency; SP: Side population

## Competing interests

The authors declare that they have no competing interests.

## Authors' contributions

ECJ, CG and DB wrote the review. All authors read and approved the final manuscript.

## Pre-publication history

The pre-publication history for this paper can be accessed here:

http://www.biomedcentral.com/1471-2407/9/202/prepub
